# NanoScope‐Assisted Arthroscopic Repair of a Full‐Thickness Supraspinatus Tear With Concomitant Shoulder Pathology: A Case Report

**DOI:** 10.1155/cro/6197200

**Published:** 2026-11-15

**Authors:** Jonathan Brutti, Juan Rayo Parra, Klaudia Greer, Zachary Grand, Jan Pieter Hommen, Cesar Ceballos

**Affiliations:** ^1^ Florida International University Herbert Wertheim College of Medicine 11200 SW 8th Street Miami 33199 Florida USA fiu.edu; ^2^ Rutgers New Jersey Medical School 185 S Orange Avenue Newark 07103 New Jersey USA rutgers.edu; ^3^ OrthoMiami 7800 SW 87th Avenue Suite A110 Miami 33173 Florida USA

**Keywords:** case report, NanoScope-assisted arthroscopy, rotator cuff repair, shoulder arthroscopy, supraspinatus tear

## Abstract

**Case:**

A 62‐year‐old right‐hand‐dominant woman presented with progressive atraumatic left shoulder pain, weakness, and limited range‐of‐motion (ROM) refractory to conservative management. Examination revealed weakness of the supraspinatus and positive impingement signs. MRI demonstrated a medially retracted full‐thickness supraspinatus tear with subacromial bursitis and acromioclavicular arthrosis. Intraoperative assessment identified a partial tear of the biceps tendon at the superior labral insertion, a degenerative Type‐II superior labrum anterior‐to‐posterior (SLAP) lesion, posterior–superior labral tearing without complete detachment, and glenoid chondromalacia. She underwent NanoScope‐assisted arthroscopic double‐row supraspinatus repair with biceps tenotomy, labral debridement, glenoid chondroplasty, subacromial decompression, acromioplasty, and distal claviculectomy. At 6 weeks, ultrasound (US) demonstrated an intact‐appearing supraspinatus repair without retraction. At 1‐year telehealth follow‐up, the patient reported favorable patient‐reported outcomes, including an American Shoulder and Elbow Surgeons score of 88/100, shoulder surgery questionnaire 8 score of 92.5/100, single assessment numeric evaluation (SANE) scores of 95% for the left shoulder and 90% for the right shoulder, and visual analog scale (VAS) pain score of 0/10.

**Conclusion:**

This case demonstrates the technical feasibility of NanoScope‐assisted arthroscopic repair of a full‐thickness supraspinatus tear with concomitant shoulder pathology.

## 1. Introduction

Rotator cuff (RTC) tears are among the most common causes of shoulder dysfunction and often require surgical intervention [1]. While conventional arthroscopy has significantly reduced morbidity compared to open repair, emerging NanoScope‐assisted arthroscopic technologies aim to further minimize tissue trauma, reduce postoperative pain, and accelerate recovery through smaller portals and reduced fluid use [2–5]. NanoScope‐assisted arthroscopy uses a chip‐on‐tip, single‐use arthroscope that enables visualization and instrumentation through 2–3 mm portals, offering reduced fluid volumes, decreased tissue disruption, and less postoperative swelling [5–8]. However, clinical evidence demonstrating superiority over standard arthroscopy, reduced pain, lower fluid use, or faster recovery remains limited.

Early literature supports the feasibility of NanoScope‐assisted arthroscopy in diagnostic applications for simple shoulder procedures, including superior labrum anterior‐to‐posterior (SLAP) tear debridement and biceps tenotomy [4]. Recent technical reports have extended its utility to RTC repair using specialized small‐bore instruments and dual‐lumen cannulas, though clinical case evidence remains sparse [2, 3]. This case describes NanoScope‐assisted arthroscopic management of a full‐thickness supraspinatus tear with concomitant biceps, labral, subacromial, chondral, and acromioclavicular pathology (Table 1). The case is presented as a technical feasibility report rather than evidence of efficacy or superiority over standard arthroscopy.

**Table 1:** Patient timeline.

Time point	Event	Key details
Initial presentation	Left shoulder pain	Progressive atraumatic left shoulder pain radiating to the arm, loss of ROM, and difficulty lifting objects. No prior left shoulder surgery.
Initial MRI	First MRI findings	Full‐thickness supraspinatus tear involving the anterior leading edge, measuring 18 × 12 mm, with 2.4 cm medial retraction. Infraspinatus, teres minor, and subscapularis tendons intact. Proximal biceps tendon intact. No rotator cuff muscle edema or atrophy. Subacromial/subdeltoid bursitis, severe AC joint arthrosis, type‐II acromion morphology, narrowed supraspinatus outlet, small glenohumeral effusion, and no labral tear.
Conservative management	Nonoperative treatment	Patient underwent conservative management with NSAIDs, physical therapy, and activity modification. Treatment was considered unsuccessful because of persistent pain, weakness, limited ROM, difficulty lifting, and imaging‐confirmed full‐thickness supraspinatus tear with retraction.
Repeat MRI, ~1 year later	Repeat MRI findings	Full‐thickness retracted supraspinatus tear again seen, now described with 2.8 cm medial retraction from the greater tuberosity insertion. Other rotator cuff tendons remained intact. No rotator cuff muscle edema or atrophy. Mild bursitis and severe AC joint arthrosis persisted. Distal clavicle and distal acromion marrow edema/small cysts were consistent with stress reaction. No labral tear. Proximal biceps tendon intact.
Preoperative evaluation	Decision for surgery	After discussion of operative and nonoperative options, the patient elected to undergo NanoScope‐assisted arthroscopic rotator cuff repair. Preoperative labs and medical clearance were completed.
Surgery	Operative treatment	NanoScope‐assisted arthroscopy was performed with double‐row supraspinatus repair, biceps tenotomy, labral debridement, glenoid chondroplasty, bursectomy, acromioplasty, subacromial decompression, and distal claviculectomy.
Postoperative day 1	Early follow‐up	Mild pain controlled with intermittent over‐the‐counter medication. Incisions were clean and intact. Neurovascular status was normal.
2 weeks postoperative	Follow‐up	Pain and swelling had resolved. Incisions were clean, dry, and intact, without erythema, drainage, or neurovascular changes.
3–4 weeks postoperative	Rehabilitation progression	Physical therapy focused on passive ROM, including pulley and pendulum exercises, according to the surgeon‐directed protocol.
6 weeks postoperative	Ultrasound follow‐up	Patient was participating in therapy. Passive ROM showed forward flexion to 135°, external rotation to 35°, and internal rotation to the lower back. Ultrasound demonstrated an intact‐appearing supraspinatus repair without retraction.
1 year postoperative	Telehealth follow‐up	Patient reported favorable outcomes: ASES 88/100, SSQ8 92.5/100, SANE 95% left shoulder and 90% right shoulder, and VAS pain score 0/10. No in‐person exam, objective strength testing, or late imaging was obtained.

This case report was prepared in accordance with the CARE reporting guidelines. Written informed consent was obtained from the patient for the publication of this case report and accompanying clinical images.

## 2. Case Presentation

A 62‐year‐old right‐hand dominant woman with no prior trauma presented with progressive left shoulder pain, radiating anteriorly and laterally, accompanied by difficulty lifting and reduced range‐of‐motion (ROM). She had a prior right RTC repair via conventional arthroscopy but no surgical history on the left. Relevant patient factors affecting tendon healing were reviewed, including smoking, diabetes status, thyroid disease, osteoporosis or osteopenia, chronic corticosteroid use, and activity level. The patient lives a sedentary lifestyle and is a nonsmoker with a BMI of 37.7 kg/m^2^. She has a medical history of essential hypertension, diabetes mellitus, and recurrent left lower extremity deep vein thrombosis that is well controlled.

On physical examination, she exhibited tenderness over the AC joint, limited active forward flexion to 150°, external rotation to 60°, and internal rotation to the midthoracic spine. Pain was provoked in the supraspinatus during external rotation resistance testing. Supraspinatus testing demonstrated pain and weakness with the empty‐can test, and resisted external rotation demonstrated 4‐/5 strength. Hawkins and Neer’s signs were positive. No instability or laxity was noted, and the distal neurovascular examination was normal.

Initial MRI of the left shoulder revealed a full‐thickness supraspinatus tear (18 mm × 12 mm) located 2.4 cm medial to its insertion. The infraspinatus, teres minor, and subscapularis tendons were intact without tear. The supraspinatus, infraspinatus, teres minor, subscapularis, and deltoid muscles demonstrated no edema or atrophy. Fluid was present in the subacromial/subdeltoid bursa, which is consistent with bursitis. The proximal biceps tendon was intact and normally positioned within the bicipital groove. Severe acromioclavicular joint arthrosis was noted, with type‐II distal acromion morphology, effacement of the subacromial fat, and narrowing of the supraspinatus outlet. A small glenohumeral joint effusion was present. A small subchondral cyst was noted in the posterolateral humeral head.

The patient underwent conservative management, including physical therapy, NSAIDs, and activity modification. Despite these measures, she continued to experience persistent left shoulder pain, weakness with lifting, limited ROM, and functional impairment. Given persistent symptoms despite nonoperative treatment, a repeat MRI was ordered to assess her left shoulder prior to further management.

A repeat MRI, approximately 1 year later from her initial imaging, demonstrated a full‐thickness retracted tear of the supraspinatus tendon, now described as 2.8 cm medial retraction from the greater tuberosity insertion. The remaining RTC tendons, including the infraspinatus, teres minor, and subscapularis, remained intact without tear. The RTC and deltoid muscles again demonstrated no edema or atrophy. Mild subacromial/subdeltoid bursitis persisted. The proximal biceps tendon remained intact and was normally positioned within the bicipital groove. Severe acromioclavicular arthrosis was again present, with effacement of the subacromial fat and narrowing of the supraspinatus outlet. The repeat MRI also described marrow edema in the distal clavicle and distal acromion with small subchondral cysts, consistent with the stress reaction. A small glenohumeral joint effusion persisted.

After reviewing operative and nonoperative options, the patient elected to proceed with NanoScope‐assisted arthroscopic RTC repair. Preoperative labs and medical clearance were completed. Two weeks before surgery, the exam findings remained consistent, with supraspinatus and external rotation weakness (4/5) and no signs of instability.

The surgery was performed under interscalene nerve block and laryngeal mask airway anesthesia. The patient was placed in lateral decubitus with 10 lbs of suspension traction. Standard anterior and posterior NanoScope‐assisted arthroscopy portals were established.

Intra‐articular assessment confirmed the full‐thickness supraspinatus tear with medial retraction (Figure 1a,b). The biceps tendon demonstrated a partial tear at its insertion site in the superior labrum, with a degenerative Type‐II SLAP lesion and tearing of the posterior–superior labrum without complete detachment. A monopolar radiofrequency probe was used to perform a biceps tenotomy, followed by debridement of the biceps stump, Type‐II SLAP lesion, and posterior–superior labral tissue with a motorized shaver. Glenoid chondroplasty was completed, and the undersurface of the cuff was debrided.

**Figure 1:**
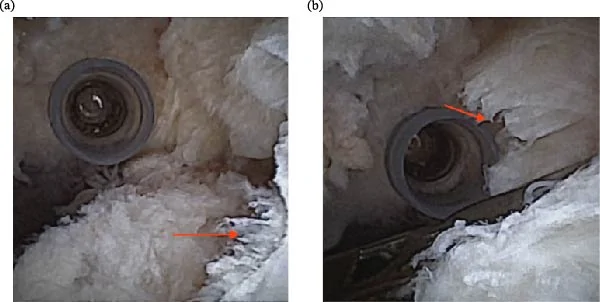
(a) Intraoperative NanoScope image of the left shoulder demonstrating the full‐thickness supraspinatus tear with medial retraction. The retracted tendon edge is marked with a red arrow. (b) Intraoperative NanoScope image demonstrating assessment of supraspinatus tendon mobility using a soft‐tissue grasper. The tendon edge is marked with a red arrow.

The subacromial space was entered via a lateral portal. Bursectomy and mobilization of the tendon were performed. The greater tuberosity was prepared with a motorized shaver to create a bleeding bone bed. A standard double‐row speed bridge repair was completed using two 2.6 mm knotless FiberTak anchors medially and two 4.75 mm biocomposite SwiveLock anchors laterally with horizontal mattress sutures (Figure 2). Fixation was confirmed. Subacromial decompression, acromioplasty, and distal claviculectomy (Mumford procedure) were also performed. All portals were irrigated and closed with layered sutures. The patient was discharged with a sterile dressing and a gunslinger sling. There were no complications.

**Figure 2:**
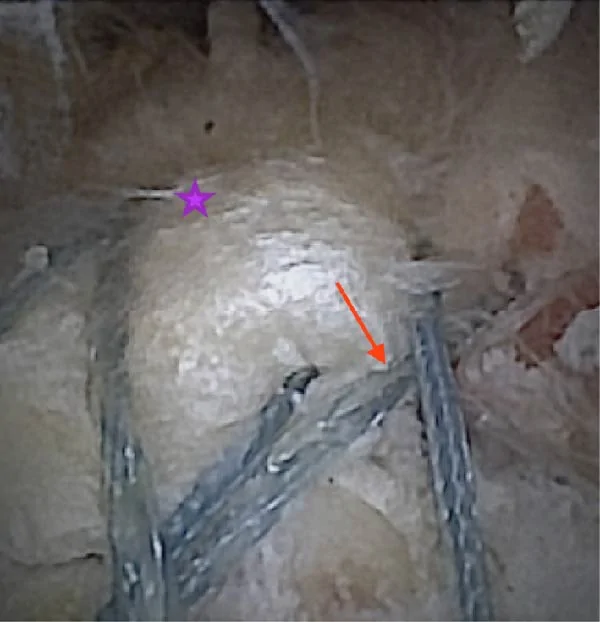
Intraoperative NanoScope image viewed from the lateral portal after double‐row supraspinatus repair. The repair line is annotated with a red arrow. The greater tuberosity footprint is marked with a purple star.

Postoperatively, the patient was placed in a sling for 4 weeks with daily removal for hygiene and elbow ROM. Passive ROM and active external rotation were initiated immediately using the Gunslinger assist device. At the initial postoperative visit (day one), the patient reported mild pain managed with intermittent over‐the‐counter (OTC) pain medication. Mild swelling and diffuse tenderness were present, but incisions were clean and intact; the neurovascular status was normal. Physical therapy was initiated 2‐3 times weekly for 6‐8 weeks following the day‐one postoperative visit. At 2 weeks postsurgery, the patient reported the resolution of pain and swelling. The incisions were clean, dry, and intact. No erythema, drainage, or neurovascular changes were noted. The patient was ordered to use a continuous passive motion machine to improve ROM as tolerated. At 3 weeks postsurgery, physical therapy focused on passive range‐of‐motion exercises, including pulley and pendulum exercises, in accordance with the surgeon‐directed rehabilitation protocol.

By 6 weeks postsurgery, she was actively engaged in therapy and reported mild activity‐related pain associated with physical therapy. Physical examination revealed well‐healed incisions, no tenderness, and passive ROM: forward flexion to 135°, external rotation to 35°, and internal rotation to the lower back. This ROM, compared to the baseline, is consistent with early healing. Mild stress testing revealed some weakness with preserved cuff activation. Ultrasound (US) confirmed an intact supraspinatus tendon without retraction, consistent with intact RTC repair (Figure 3a). Furthermore, US imaging detailed supraspinatus muscle fibers attaching to the humeral greater tuberosity (Figure 3b). Postoperative US was performed in the office by the physician associate. The images were subsequently interpreted by the orthopedic surgeon who performed the surgery. Additional technical details regarding the US examination were not available retrospectively.

**Figure 3:**
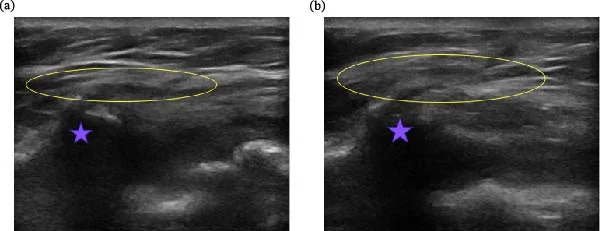
(a) Six‐week postoperative ultrasound image of the left shoulder demonstrating supraspinatus fibers attaching to the humeral greater tuberosity. The supraspinatus tendon repair is highlighted with a yellow oval and the greater tuberosity with a purple star. (b) Six‐week postoperative coronal ultrasound image demonstrating an intact‐appearing supraspinatus repair without retraction. The repaired tendon is marked with a yellow oval, and the greater tuberosity is marked with a purple star.

During a follow‐up telehealth visit with the patient 1 year after surgery, she reported significant improvement in pain and function in her left shoulder. At this time, her ASES shoulder score was 88, with higher scores indicating better shoulder function, and the SSQ8 score was 92.5, with higher scores indicating greater satisfaction with the surgical outcome. Additionally, she self‐reported her left shoulder as 95% normal and her right shoulder as 90% normal using a single assessment numeric evaluation (SANE). Furthermore, she self‐reported a visual analog scale (VAS) score of 0/10, indicating no current pain at the time of conversation.

Complications were assessed through available postoperative visits and patient‐reported telehealth follow‐up. No intraoperative complication, infection, wound drainage, erythema, portal‐site complication, neurovascular deficit, or stiffness requiring intervention was documented. At the available follow‐up, the patient reported no persistent pain. After biceps tenotomy, she denied Popeye deformity, cramping, or cosmetic concern. However, the final complication assessment was limited by the telehealth nature of the 1‐year follow‐up and the absence of late postoperative imaging.

## 3. Discussion

Traditional arthroscopy has long been the standard for managing RTC pathology, offering clear intra‐articular visualization and the ability to address multiple structures in a single procedure [9, 10]. This case demonstrates that NanoScope‐assisted arthroscopy can be used to perform double‐row supraspinatus repair and address concomitant biceps, labral, subacromial, chondral, and AC joint pathology in a single operative setting. However, this report should be interpreted as evidence of technical feasibility only. The procedures performed in this case are routinely feasible with standard arthroscopy, and this single uncontrolled case cannot demonstrate that NanoScope‐assisted arthroscopy improves clinical outcomes, reduces pain, lowers opioid use, decreases irrigation fluid volume, or accelerates recovery. Although the patient reported only intermittent OTC analgesic use early postoperatively, this observation cannot be attributed specifically to NanoScope‐assisted arthroscopy. Future comparative studies should evaluate postoperative pain scores, opioid consumption, swelling, irrigation volume, functional outcomes, and tendon healing after NanoScope‐assisted versus standard arthroscopic RTC repair.

While prior shoulder reports have mainly described NanoScope‐assisted arthroscopy for diagnostic applications, isolated pathology, or technical procedures [2–5, 11], this case report contributes to this literature by describing NanoScope‐assisted management of a full‐thickness supraspinatus tear with concomitant AC joint arthrosis, subacromial bursitis, glenoid chondromalacia, degenerative Type‐II SLAP pathology, posterior–superior labral tearing, and a partial biceps tendon tear at its superior labral insertion.

Recent literature further supports the need to study NanoScope‐assisted arthroscopy as an evolving therapeutic platform [12]. Wojtowicz et al. [13] described a NanoScope‐assisted arthroscopic repair of an acute tibiofibular syndesmosis injury, illustrating the use of NanoScope‐assisted arthroscopy as an adjunct in therapeutic orthopedic procedures. A cadaveric ankle study evaluating NanoScope‐assisted arthroscopy emphasized visualization, feasibility, and technical considerations, highlighting that procedure‐specific validation and surgeon familiarity remain important [14]. In the shoulder, Lesman et al. [15] reported NanoScope‐assisted long‐head biceps tendon tenotomy under suprascapular nerve block as a feasible approach in a prospective feasibility study. These studies provide useful context for the expanding therapeutic use of NanoScope‐assisted techniques, but they also reinforce the need for larger comparative studies before conclusions can be drawn regarding superiority, postoperative pain, or functional recovery.

One particularly noteworthy aspect of this technique is its reduced requirement for irrigation fluid [8, 16]. A reduced portal size and potentially reduced irrigation requirements are proposed advantages of NanoScope‐assisted arthroscopy, but these variables were not objectively measured in this case. Standard arthroscopy typically involves copious lavage to maintain visualization and joint distention, but excessive fluid pressure can contribute to postoperative swelling, joint overexpansion, and delayed recovery [16]. At a 1‐year telehealth follow‐up, the patient reported a SANE score of 95% for the operative left shoulder and 90% for the contralateral shoulder previously treated with conventional arthroscopy. This comparison is patient‐reported, uncontrolled, and subject to recall and treatment‐selection bias; therefore, it should be interpreted cautiously. While these findings are encouraging, conclusions cannot be drawn from a single case, and future comparative studies are needed to establish causality and determine whether patient‐reported outcomes are comparable between techniques.

In summary, this case illustrates the technical feasibility of NanoScope‐assisted arthroscopic repair of a full‐thickness supraspinatus tear with concomitant shoulder pathology. However, the findings are hypothesis‐generating and do not establish efficacy, superiority, reduced pain, lower fluid use, or faster recovery compared with standard arthroscopy. Further comparative studies using standardized preoperative and postoperative outcome measures, objective physical examination, imaging follow‐up, and systematic complication assessment are needed.

### 3.1. Limitations

This report has several limitations. First, it describes a single patient without a control group, precluding conclusions regarding efficacy or superiority compared with standard arthroscopy. Second, preoperative patient‐reported outcome scores were unavailable, preventing direct comparison with the 1‐year postoperative ASES, SSQ8, SANE, and VAS scores. These results should therefore be interpreted as favorable postoperative patient‐reported outcomes rather than objective evidence of improvement from baseline.

Third, the 1‐year follow‐up was conducted by telehealth, which precluded objective range‐of‐motion measurement, strength testing, physical examination, and systematic assessment for biceps tenotomy‐related complications. No late MRI or US was obtained, so long‐term tendon integrity could not be confirmed. Fourth, multiple concomitant procedures were performed, including biceps tenotomy, labral debridement, glenoid chondroplasty, subacromial decompression, acromioplasty, and distal claviculectomy, preventing attribution of the postoperative outcome specifically to NanoScope‐assisted arthroscopy.

Additionally, total irrigation volume, pump pressure, cannula type, and portal incision size were not documented in the operative report; therefore, reduced fluid use cannot be inferred from this case. Practical limitations of NanoScope‐assisted arthroscopy also include cost, accessibility, the learning curve, instrument limitations, and field‐of‐view constraints. Accordingly, this case demonstrates technical feasibility but does not establish clinical superiority or accelerated recovery.

## Author Contributions

Jonathan Brutti, Juan Rayo Parra, Klaudia Greer, and Zachary Grand drafted the initial version of the manuscript. Cesar Ceballos performed the surgical treatment. Jonathan Brutti, Juan Rayo Parra, Klaudia Greer, and Zachary Grand conducted patient follow‐up and collected the data. Jan Pieter Hommen and Cesar Ceballos supervised the entire process. Jonathan Brutti and Juan Rayo Parra conducted revisions and edits of the manuscript following initial submission.

## Funding

No funding was received for the purposes of this manuscript.

## Ethics Statement

This case report was conducted in accordance with the ethical principles outlined in the Declaration of Helsinki. Written informed consent was obtained from the patient for the publication of this case report and accompanying clinical images. Institutional review board approval was not required for a single‐patient case report according to the institutional policy.

## Conflicts of Interest

The authors declare no conflicts of interest.

## Patient Perspective

At a 1‐year telehealth follow‐up, the patient reported substantial satisfaction with the surgical outcome, no current pain, and favorable self‐reported shoulder function.

## Data Availability

Data sharing is not applicable to this study as no datasets were utilized, generated, or analyzed.
